# Supplementary material for: A Critical View of the Application of the APEX Software (Aqueous Photochemistry of Environmentally-Occurring Xenobiotics) to Predict Photoreaction Kinetics in Surface Freshwaters
Source: Molecules. 2019 Dec 18;25(1):9. doi: 10.3390/molecules25010009 (PMC7017383; doi:10.3390/molecules25010009)
Supplement: Supplementary file 1 [file molecules-25-00009-s001.zip › 3.2.4_gcc-4.4.0.zip!/3.2.4_gcc-4.4.0/3.2.4_gcc-4.4.0/doc/octave/html/interpreter/Returning-From-a-Function.html]

Returning From a Function - Untitled


Next: Default Arguments,
Previous: Variable-length Return Lists,
Up: Functions and Scripts


---

### 11.5 Returning From a Function

The body of a user-defined function can contain a `return` statement.
This statement returns control to the rest of the Octave program. It
looks like this:

```
     return
```

Unlike the `return` statement in C, Octave's `return`
statement cannot be used to return a value from a function. Instead,
you must assign values to the list of return variables that are part of
the `function` statement. The `return` statement simply makes
it easier to exit a function from a deeply nested loop or conditional
statement.

Here is an example of a function that checks to see if any elements of a
vector are nonzero.

```
     function retval = any_nonzero (v)
       retval = 0;
       for i = 1:length (v)
         if (v (i) != 0)
           retval = 1;
           return;
         endif
       endfor
       printf ("no nonzero elements found\n");
     endfunction
```

Note that this function could not have been written using the
`break` statement to exit the loop once a nonzero value is found
without adding extra logic to avoid printing the message if the vector
does contain a nonzero element.

— Keyword: **return**  
> When Octave encounters the keyword `return` inside a function or
> script, it returns control to the caller immediately. At the top level,
> the return statement is ignored. A `return` statement is assumed
> at the end of every function definition.
